# Nanomedicine in fungal keratitis: current applications and future prospects

**DOI:** 10.3389/fmicb.2025.1618046

**Published:** 2025-07-09

**Authors:** Yuyang Xiao, Yifei Yang, Binyu Sun, Meng Yang, Jiamiao Lang, Mintao Dong, Zengsihan Chen, Shanshan Chen, Shengfeng Wang

**Affiliations:** ^1^Department of Pharmacy, Hunan Cancer Hospital/The Affiliated Cancer Hospital of Xiangya School of Medicine, Central South University, Changsha, China; ^2^Xiangya School of Medicine, Central South University, Changsha, China; ^3^Department of Pharmacy, The Third Xiangya Hospital, Central South University, Changsha, China

**Keywords:** fungal keratitis, nanomedicine, drug delivery system, treatment, bibliometric analysis

## Abstract

Fungal keratitis (FK) poses a significant public health challenge, causing substantial harm to human health and the socio-economic landscape. However, due to the special anatomical and physiological characteristics of the eye, the current therapeutic drugs for FK are not effective, which brings obstacles to the clinical treatment of FK. Nanomedicine is a new therapeutic method that has emerged in the field of FK therapy in recent years. On the one hand, nanomaterials can directly kill fungi by separating ions, and on the other hand, the drug delivery system (DDS) composed of nanomaterials can target and deliver traditional drugs to the affected area and play a bactericidal role, with a significantly higher effect than traditional therapy. This review begins with a bibliometric analysis of research progress in FK nanomedicine. Then we describe the mechanism and effect of various nanomedicine in the treatment of FK from the perspective of direct and indirect treatment, focusing on the attack of nanomedicine on biofilm and DDS composed of nanomaterials. Finally, this field is prospected in order to provide new insights and ideas for the development of FK nanomedicine.

## 1 Introduction

Fungal keratitis (FK) is an infectious form of keratitis primarily caused by filamentous fungi such as Aspergillus and Fusarium, as well as yeast-like fungi such as Candida (Spierer et al., 2015; Mishra et al., 2020). Currently, more than 100 fungal species have been identified as potential causes of FK (Donovan et al., 2022). The incidence of infectious keratitis is on the rise (Ung et al., 2019), with FK accounting for approximately 45% of the 1 million new corneal infections reported annually (Niu et al., 2020). It is estimated that there are 1 million cases of FK globally each year, and more than half of the patients with FK may lose their vision and suffer from monocular blindness (Brown et al., 2021). This condition is particularly prevalent in developing countries in Asia and Africa (Bisen et al., 2024), where it poses a significant public health challenge. Compared to bacterial keratitis (BK), the diagnosis of FK is often delayed, and treatment options are limited. As a result, clinical outcomes and prognoses for FK tend to be worse than those for BK (Prajna et al., 2012). Mild cases of FK can progress to severe corneal disease (Kern, 1990; Whitcher and Srinivasan, 1997; Garg and Rao, 1999). FK frequently manifests alongside corneal ulcers, a condition referred to as ulcerative fungal keratitis (UFK) (Anderson et al., 1959). UFK tends to be particularly serious (Whitcher and Srinivasan, 1997; Brown et al., 2022) and can result in permanent visual impairment or blindness.

**Figure 1:**
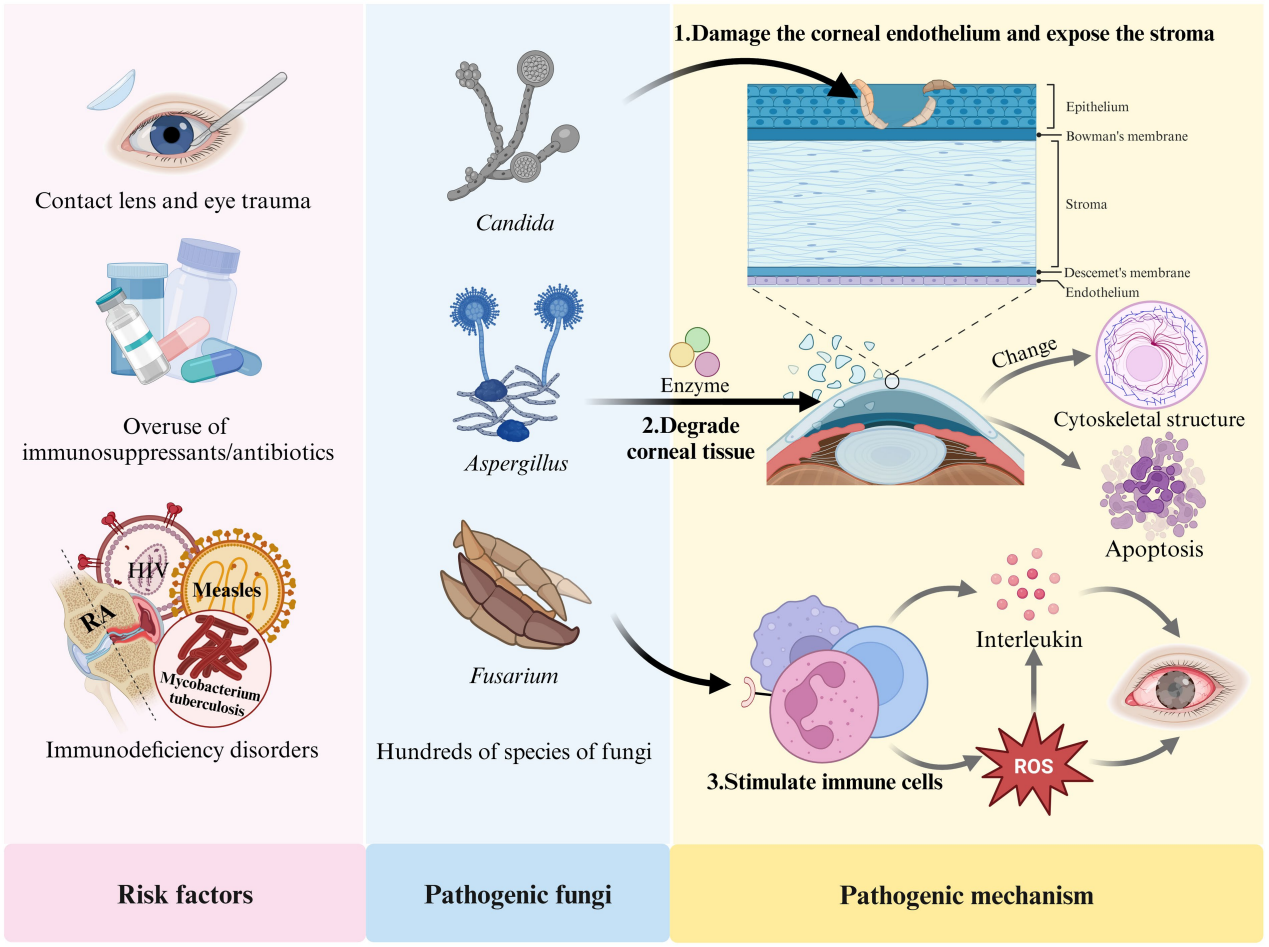
Risk factors, pathogenetic fungi and pathogenic mechanism of FK.

The development of FK is closely associated with several factors, including prolonged contact lens wear, eye trauma, the overuse of immunosuppressants or broad-spectrum antibiotics, eye surgeries (such as corneal transplantation), and immunodeficiency diseases (including acquired immunodeficiency syndrome (AIDS), diabetes, and systemic immunodeficiency disorders) (Jin et al., 2022; Ler et al., 2022; Reginatto et al., 2023; Stapleton, 2023). FK results from the interaction between fungi and their host. Fungi can damage the human corneal epithelium, exposing the basement membrane, which allows the fungus to attach to the extracellular matrix of the corneal tissue. The fungi then produce enzymes that degrade the tissue, facilitating the spread of mycelia within the cornea. Fungal attachment enhances the transmembrane signaling mechanisms and alters the cytoskeletal structure of host cells, leading to the rearrangement of microtubules and microfilaments, ultimately resulting in apoptosis (Wu et al., 2016; Sitnova and Svetozarskiy, 2023). In response to fungal invasion, the host activates its defense mechanisms; pattern recognition receptors stimulate neutrophils to secrete various interleukins and promote the production of reactive oxygen species (ROS) (Abbondante et al., 2023). However, excessive ROS can lead to an overproduction of IL-1β in the body (Liu et al., 2023), causing eye tissue damage and promoting fungal growth, which further exacerbates FK. Figure 1 illustrates the risk factors, pathogenic fungi, and pathogenic mechanisms associated with FK.

Currently, the drug treatment for FK primarily focuses on the combination of antifungal and anti-inflammatory medications (Manzouri et al., 2001; Lakhani et al., 2019; Fisher et al., 2022). This includes local applications of natamycin (NATA), amphotericin B, fluconazole, and voriconazole, often combined with dexamethasone and non-steroidal anti-inflammatory drugs (Shen et al., 2011; Hou et al., 2023; Mishra et al., 2024). Amphotericin B is frequently used as a first-line treatment for FK (Ansari et al., 2013), while topical NATA eye drops are the preferred option for filamentous FK (Mahmoudi et al., 2018; Rohira et al., 2021). At present, there are no commercially available amphotericin B eye drops, and the amount of the drug that reaches the affected area is low (Diebold et al., 2007), necessitating frequent long-term administration. Similarly, NATA eye drops have a short residence time in the cornea (Gu et al., 2022) and require frequent application. Prolonged medication can exacerbate the underlying condition and/or lead to complications, posing challenges for patient compliance (Chandasana et al., 2014; Chhonker et al., 2015; Bhattacharya et al., 2020; Vitiello et al., 2023). Furthermore, antifungal drugs are susceptible to developing resistance, which can result in treatment failure (Wiederhold, 2017; Kaul et al., 2022). Although new antifungal agents such as posaconazole (Ferguson et al., 2022) and esaconazole (Cultrera et al., 2021) have been introduced, but their therapeutic effects remain limited. Study suggests that the use of traditional Chinese medicine in combination with antifungal drugs can enhance therapeutic efficacy and reduce drug side effects (Huang et al., 2022). In addition to the aforementioned medications, research on surgical treatments for FK, such as corneal transplantation (Thomas and Kaliamurthy, 2013), photodynamic therapy (Amescua et al., 2017; Hung et al., 2021) and sutureless corneal adhesion (Choi and Jeon, 2022), is also increasing. However, these methods all have certain limitations and drawbacks. There is an urgent need to explore innovative therapeutic approaches for FK.

Nanomaterials are particles with diameters ranging from 1 to 100 nm, characterized by high specific surface areas and adjustable physical and chemical properties. They have found widespread applications in various medical fields, including cancer treatment, drug delivery, diagnostic imaging, antibacterial therapy, burn treatment and wound healing (Aflori, 2021; Kakodkar et al., 2023). Metal nanoparticles and metal oxide nanoparticles exhibit broad-spectrum antimicrobial activity, and certain nanomaterials have demonstrated antibacterial effects against filamentous fungi such as Aspergillus, Coccidioides, and Mucor (Leon-Buitimea et al., 2021). Furthermore, nanomaterials can serve as carriers for drug transport. Drug molecules can bind to nanoparticles, resulting in the formation of coupled nanoparticle-drug complexes. Drug molecules can be loaded into nanoparticles by following methods: attachment to the surface, conjugation, entrapment, and encapsulation (Liu et al., 2012; Achouri et al., 2013). Solid lipids encapsulated by nanoparticles are referred to as lipid nanocarriers (Gu et al., 2022). A drug delivery system (DDS) composed of nanomaterials for FK can enhance the residence time of drugs in the cornea (Liu et al., 2012), improve bioavailability and stability, ensure continuous and controlled release, and provide targeted delivery to specific ocular tissues (Kakodkar et al., 2023). Compared to traditional treatment methods, nanopreparations can significantly enhance the quality of medical treatment, offering higher efficacy with fewer adverse reactions, thus demonstrating substantial clinical therapeutic potential.

This review utilizes bibliometrics to analyze the current status and progress in the field of nanomedicine therapy for FK in recent years. It begins by introducing the anatomical and physiological characteristics of the eye. We then discuss the advancements in nanomaterials for the treatment of FK from both direct and indirect therapeutic perspectives. Additionally, we explore the mechanisms and properties of various nanomaterials used in FK treatment, with a particular focus on the action of nanoparticles against biofilms and the DDS involved in FK therapies. Finally, we present prospects for future development in this field, aiming to investigate the clinical application value of nanomaterials in treating FK.

## 2 Bibliometric analysis in nanomedicine and FK

Bibliometrics is an emerging method of literature analysis that systematically evaluates publications within a specific field. By statistically analyzing the characteristics and trends of papers published in this area, bibliometrics examines relationships between years, countries and regions, and keywords (Lin et al., 2022). This approach aims to reveal research progress and predict future development trends, providing valuable insights for researchers and guiding investigations within the field (Lin et al., 2020). On August 18, 2024, we conducted a search in the Web of Science Core Collection (WoSCC) database and successfully obtained 105 publications. Our retrieval method utilized the following search string: (fungals OR fungal OR fungi) AND (keratic OR keratitis OR keratitides) AND (nanomedicinal OR nanomedicine OR nanomedicines OR nanostructure OR nanostructures OR nanomaterial OR nanomaterials OR nanopreparation OR nanopreparations OR nanoparticles OR nanoparticle). We focused on articles and reviews, manually screening to retain 100 relevant studies related to FK and nanomedicine. To avoid the influence of subjective factors on the screening results, the screening process was independently completed by three authors. If there were any discrepancies in the results, discussions were held until all three authors reached a consensus and obtained the same screening results. Complete records for each publication, along with cited references, were exported. Subsequently, we employed VOSviewer, CiteSpace 6.2. R4, and Microsoft Office Excel for data statistics and visual analysis. Our analysis included the annual number of published papers, keyword co-occurrence, keyword evolution over time, and country co-occurrence.

**Figure 2:**
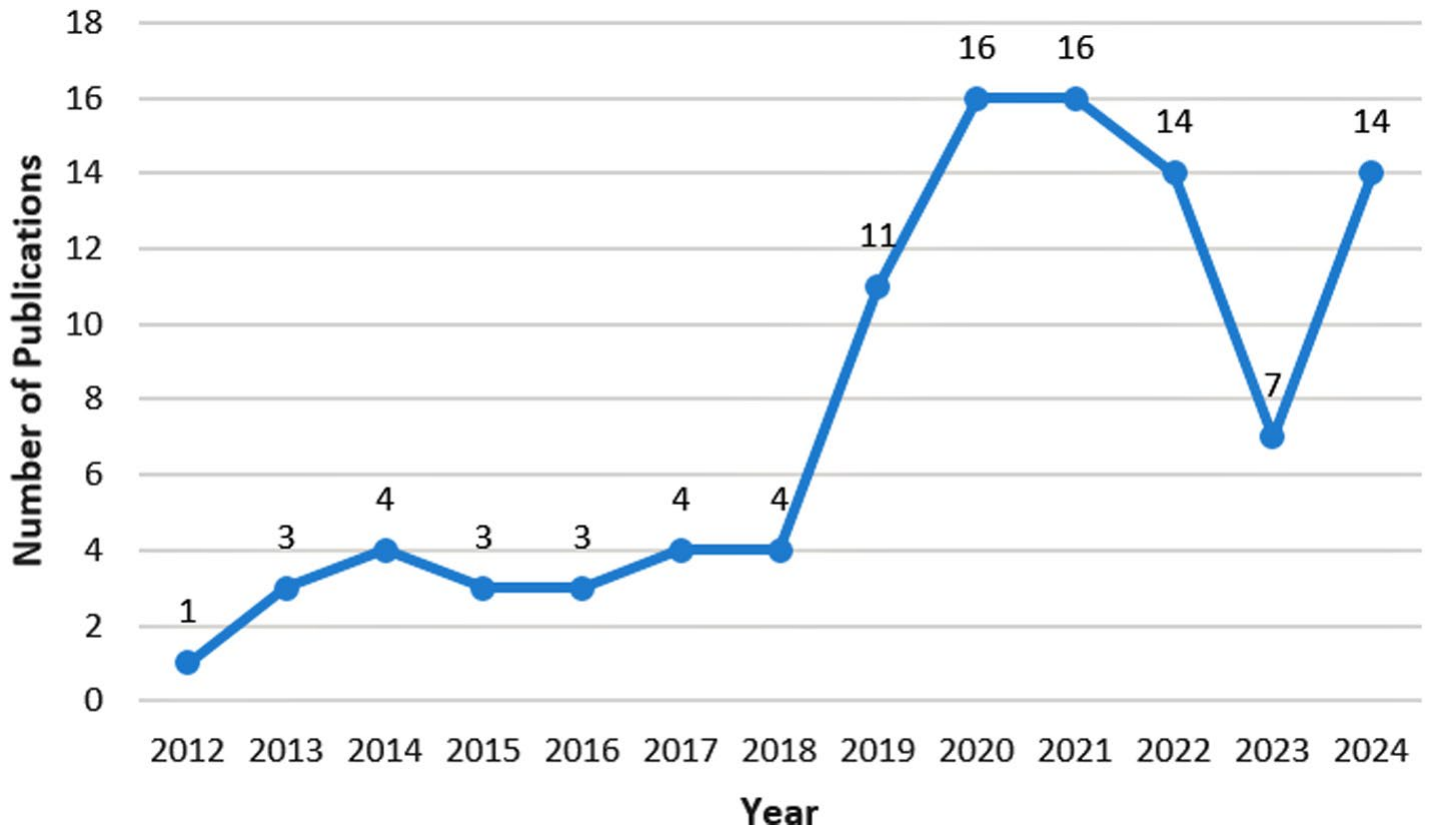
Year distribution of the 100 articles.

As nanomedicine has only emerged in the treatment of FK in recent years, the number of relevant articles is relatively small (Figure 2). There were only relevant articles from 2012, and merely 100 articles were retrieved by August 2024. Since 2018, this research field has received attention with an exploding growth in the number of studies. The field declined in popularity in 2023, but gained traction again in 2024. The most cited paper investigates a hybrid hydrogel-based contact lens, which comprises quaternized chitosan (HTCC), silver nanoparticles, and graphene oxide (GO). This contact lens can encapsulate voriconazole and may be a promising method for the rapid and effective treatment of fungal keratitis (Huang et al., 2016). The second most cited paper examines lecithin/chitosan nanoparticles that can encapsulate amphotericin B, thereby prolonging its residence time on the cornea and treating fungal keratitis (Chhonker et al., 2015). The third most cited paper explores NATA solid lipid nanoparticles (NATA-SLNs). These nanoparticles extend the drug release rate, enhance corneal permeability, increase antifungal activity, and exhibit no cytotoxic effects on corneal tissue (Khames et al., 2019).

**Figure 3:**
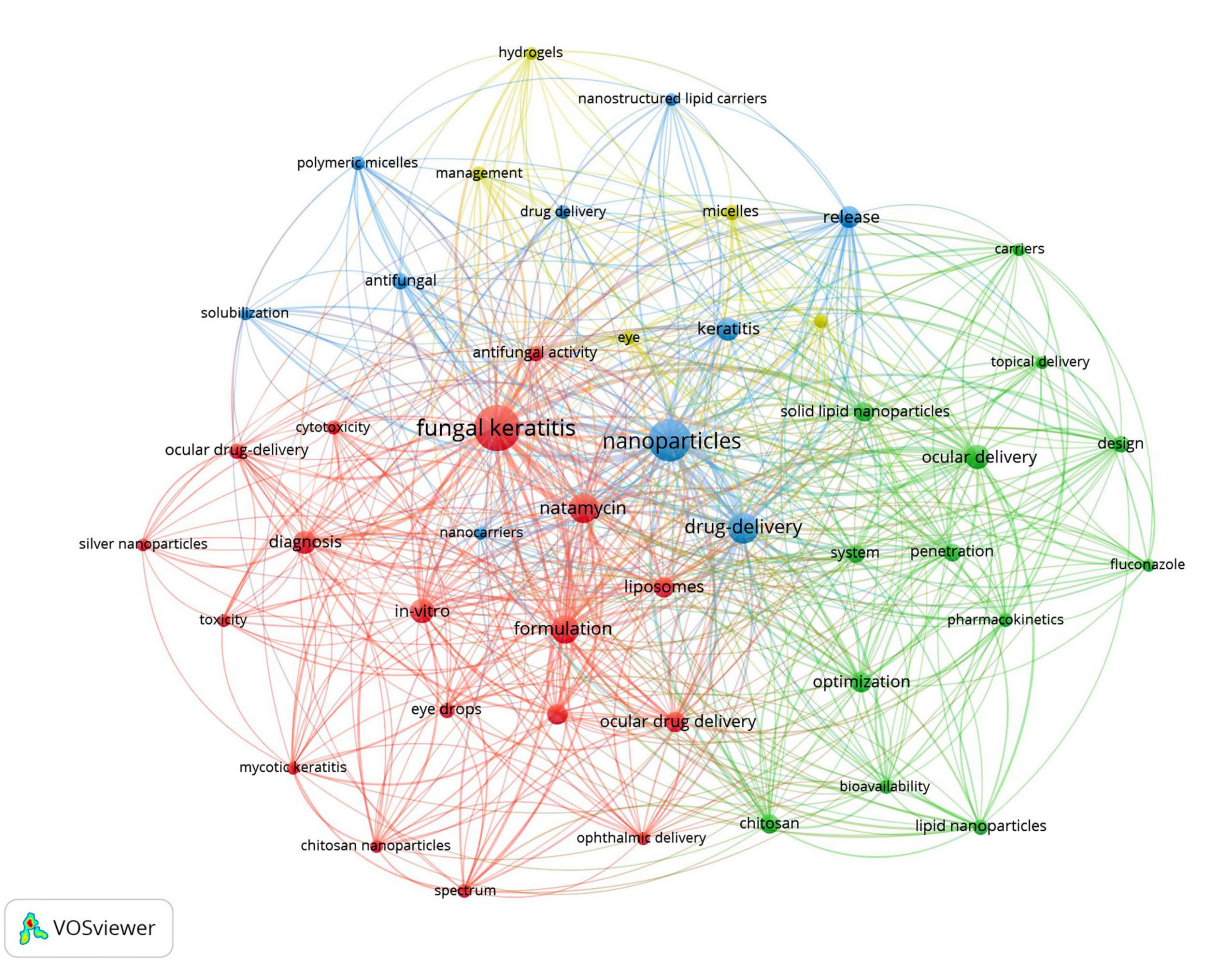
The co-occurrence network of keywords.

**Figure 4:**
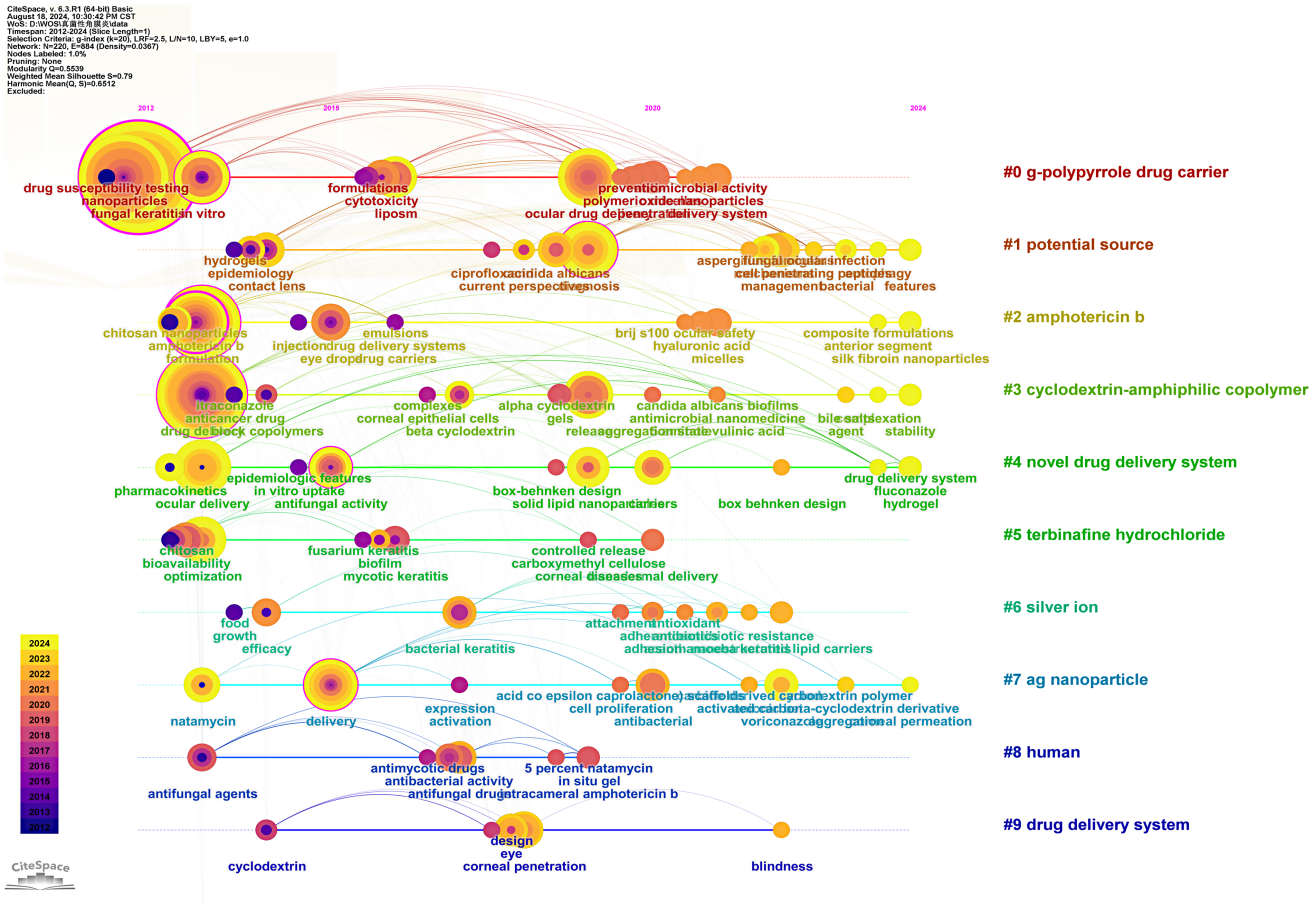
Evolution of keywords temporal trends.

We utilized VOSviewer to extract and analyze keywords, retaining a total of 46 keywords after screening. The visual analysis of keyword clustering is presented in Figure 3. Different colors in the figure represent distinct clusters, while larger node labels indicate more frequent appearances of the corresponding keywords. The top three keywords by frequency are “fungal keratitis,” “nanoparticles,” and “drug delivery,” respectively. We also examined the evolutionary trends of these keywords over time, with the results depicted in Figure 4.

**Figure 5:**
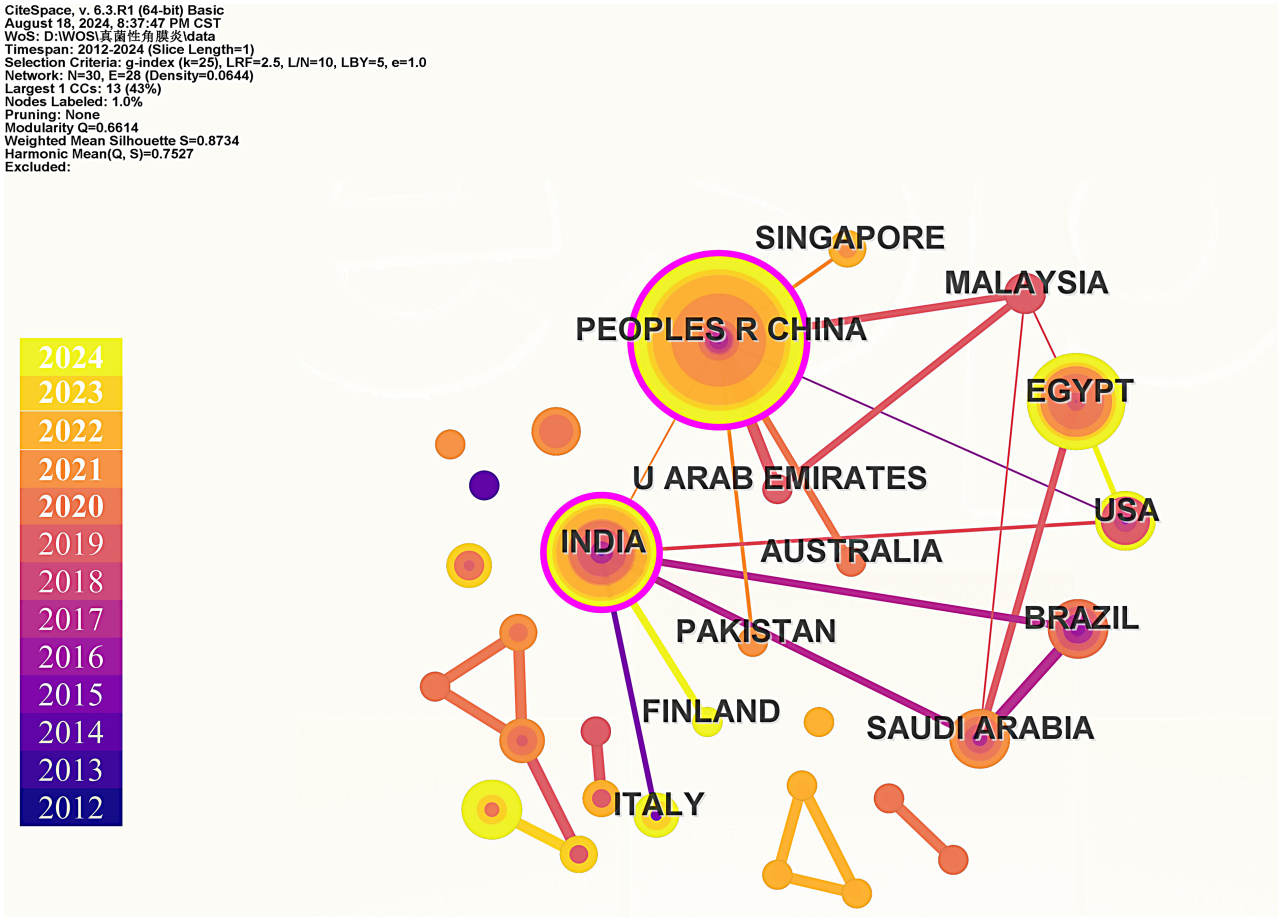
Collaboration analysis of countries/regions.

Finally, we used CiteSpace to construct the cooperation network graph among countries/regions (Figure 5). China has published the most papers (39), followed by India (19) and Egypt (17). The largest connected component for a specific country/region consists of 30 nodes and 28 connections, with a map density of 0.0644. After calculating the betweenness centrality, the top three countries identified are China, India, and Malaysia, with both China and India exhibiting betweenness centrality values greater than 0.1. This indicates that these countries/regions play significant roles in research within this field. According to the statistical results presented, China emerges as the core country in this area of study.

## 3 Anatomical and physiological characteristics of the eye

**Figure 6:**
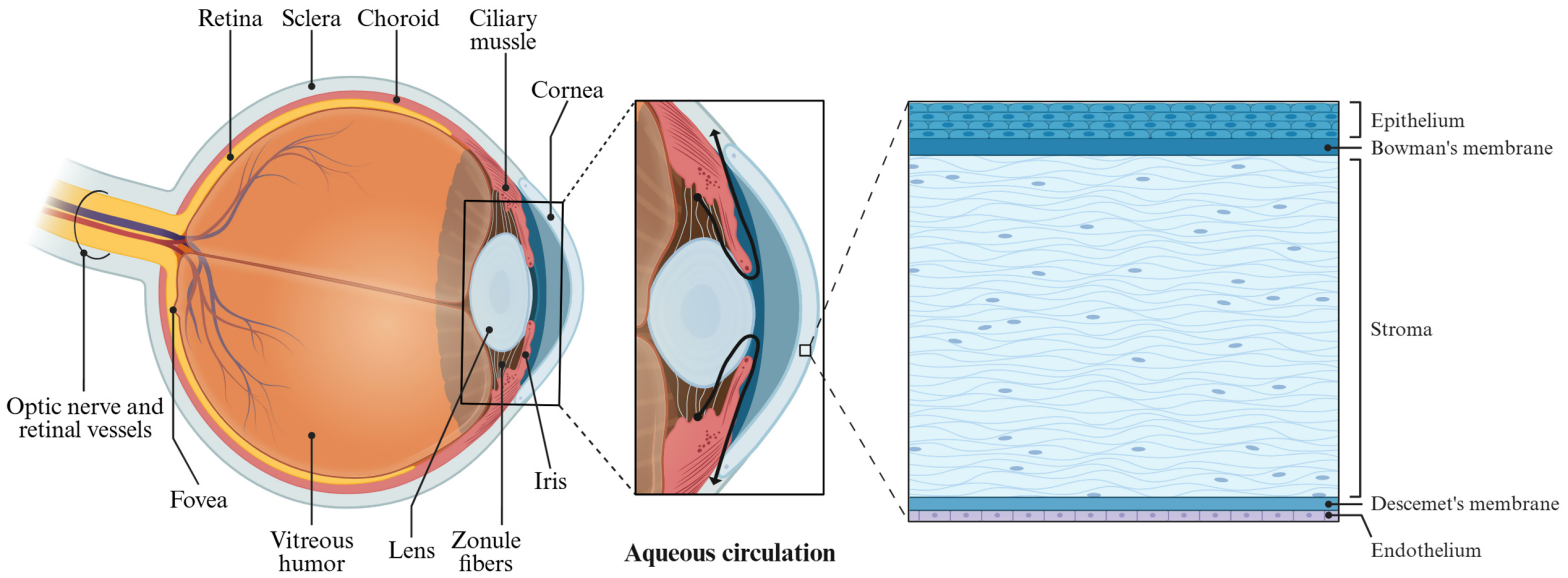
Structure of eyeball, cornea, and circulation of aqueous humor.

The eyeball is composed of the sclera, cornea, and its internal contents. The primary structures are situated within the eye’s framework, with the optic nerve at the rear connecting to the brain (Figure 6). The anatomy of the eyeball consists of two main parts: the eyeball wall and its contents. The eyeball wall can be further divided into three layers: the fibrous layer, the vascular layer, and the retina. The internal contents of the eyeball include the lens, vitreous body, and aqueous humor (Baino and Kargozar, 2020; MercuȚ et al., 2020; Xu et al., 2022). The anterior portion of the eyeball wall is the cornea, which constitutes approximately one-fifth of the total structure. The cornea is a transparent refractive surface characterized by its convex shape and lack of blood vessels, although it is densely packed with nerve endings (Thomasy et al., 2014).

In microanatomy, the cornea is composed of five layers: the epithelial cell layer, anterior elastic layer, interstitial layer, posterior elastic layer, and endothelial cell layer (Figure 6). The epithelial cells of the cornea create tight junctions that help prevent foreign objects and drugs from penetrating. Additionally, a tear film coats the surface of the corneal epithelial layer, providing a barrier function that inhibits drug absorption. As a result, conventional ophthalmic eye drops face challenges in the effectively delivering of therapeutic agents to the eye.

Normal physiological activities in humans, such as tear production, blinking and eye tissue metabolism, can significantly influence the drug delivery of Enhanced Filamentous Growth Protein 1 (EFG1) (Patel et al., 2018). The human eyeball possesses both dynamic and static barriers to drug penetration. The static barriers include the corneal epithelial cells and stroma, while the dynamic barriers consist of conjunctival blood flow, lymphatic circulation, and tear flow. These barriers collectively hinder drugs from entering the anterior chamber of the eye (Pal et al., 2013). The anterior chamber contains aqueous humor, which is produced by the ciliary body and serves to nourish the cornea. Aqueous humor circulates from the posterior chamber into the anterior chamber through the pupil (Figure 6). In systemic drug administration (Goel et al., 2010), drugs are delivered to the cornea via this aqueous humor circulation. Under physiological conditions, the typical tear flow rate in humans is approximately 1.2 mL/min, with the tear film being replenished about every 5 min. However, during tear production, this flow rate can increase dramatically to 300 mL/min, resulting in the rapid washing away of drugs. Moreover, the tear film features a hydrophilic layer formed by mucin, which protects the eyeball from foreign objects but also presents a barrier to drug absorption (Mannermaa et al., 2006).

Due to the anatomical and physiological barriers present in the eyeball, it is challenging for traditional therapeutic agents for FK to remain in the eye and achieve effective absorption. Consequently, there is an urgent need to develop new drug formulations that can enhance the therapeutic efficacy for treating FK.

## 4 Ophthalmic nanodrug delivery mechanism

Traditional ophthalmic medication uses eye drops, but due to the special anatomical and physiological structure of the eye, it is difficult for the drug to stay for a long time. At the same time, FK often occurs in the form of chronic diseases and needs to be administered frequently (Nayak and Misra, 2018). Nanocarriers are used to ensure drug targeting and controlled drug release.

Nanocarriers have specific surface charges, which are conducive to their conjugation and retention at specific sites. This potential is measured as zeta potential (Patra et al., 2018). Zeta potential refers to the potential difference between the surface charge of the nano carrier and the opposite charge from the medium arranged around the particles. Zeta potential reflects the stability of the nano carrier. According to the principles of physics, if two particles have high zeta potential of the same charge, they will repel each other because of the repulsive force, preventing the aggregation of particles. The surface of the human cornea carries a negative charge. Therefore, cationic nanoparticles will be attracted to the cornea due to electrostatic forces, which enables the cationic nanoparticles to remain on the cornea (Tsai et al., 2018). This is the mechanism by which nano carriers deliver drugs to the anterior eye region.

Ophthalmic nanodrugs can achieve targeted delivery of drugs, thereby reducing the required drug concentration and reducing the occurrence of side effects. At the same time, nanomaterials have a certain adhesion effect. Nanoparticles adhere to the surface of fungi through the action of electric charge, cutting off some key enzyme pathways and damaging the integrity of the membrane. At the same time, some nano materials have certain antifungal effect, which can reduce the fungal resistance of drugs (Guo and He, 2024).

## 5 Manufacturing process of ophthalmic nanomaterials

Ophthalmic nano materials can be divided into lipid based nano carriers, polymer nano carriers and inorganic nano carriers according to their materials (Guo and He, 2024). Lipid nanocarriers include liposomes, niosomes, solid lipid NPs, nanostructured lipid carriers and microemulsion. Polymer nanocarriers include dendrimers, micro micelles, hydrogel, chitosan nanoparticles. Inorganic nano carriers include carbon nanotubes, metal particles.

### 5.1 Lipid nanocarrier

Lipid nanocarrier is composed of phospholipid, lipid coupling polymer and cholesterol. The diameter of lipid nanocarrier varies according to different manufacturing processes. Phospholipid bilayer is lipophilic and hydrophilic, and can contain water-soluble or lipid soluble drugs (Sha et al., 2021). Niosomes are self-assembled vesicles of nonionic surfactants in water. The hydrophobic tail of the surfactant monomer is hidden in the central aqueous phase space of the double-layer vesicle structure, while the hydrophilic head group keeps in contact with them, which together constitute the double-layer structure of niosome (Sha et al., 2021). Microemulsion is a spherical colloidal structure, mainly including o/w (oil in water) and w/o (water in oil) structures. It is prepared by dispersing oil droplets in the water phase or dispersing water droplets in the oil phase (Raj et al., 2021). Solid lipid nanoparticles (SLNs) are synthesized from lecithin, triacylglycerol, active drug molecules and surfactants. Its preparation process is to encapsulate or embed the active ingredients in the lipid like core to form colloid (Rajpoot, 2019). On the basis of SLNs, researchers designed nanostructured lipid carrier (NLC), which is composed of biological lipids (Patel and Patel, 2021).

### 5.2 Polymer nanocarrier

Polymer nanocarriers are composed of natural or synthetic polymers, which can be divided into nanospheres or nanocapsules, and can carry drugs. Dentrimers are nanoscale macromolecules, which show dendritic structure in the ultrastructure and are composed of high molecular polymers of carbon or silicon (Mahaling et al., 2023). Micro mices have a core-shell structure and are formed by self loading of diblock or multiblock amphiphilic molecules (Qamar et al., 2019). Hydrogel is formed from hydrophilic polymers. Natural or synthetic polymers form hydrogel after absorbing a large amount of water (Li and Mooney, 2016).

### 5.3 Inorganic nanocarrier

Researchers used mesoporous carbon (meso-C) or microporous carbon (Micro-C) to load drugs, so as to achieve the effect of targeted therapy. Calcium alginate is obtained by using sodium alginate solution and dropping calcium chloride solution through the needle. After freeze drying, heating, soaking in hydrochloric acid and ultrasonic treatment of calcium alginate, most of the calcium ions were replaced by hydrogen ions to obtain meso-C (Gu et al., 2022).

Liu et al. developed contact lenses carrying silver nanoparticles, and used dopamine and bio gel to attach silver nanoparticles to contact lenses. The photothermal effect of gold nanoparticles is helpful for the treatment of FK. In order to prepare Rose Bengal polypyrrole gold NP complex, the researchers loaded RB on gold nanoparticles. When illuminated, RB produced reactive oxygen and gold nanoparticles produced thermal energy. This photothermal therapy helps eliminate fungi (Ghoniem et al., 2023).

## 6 Direct treatment

**Figure 7:**
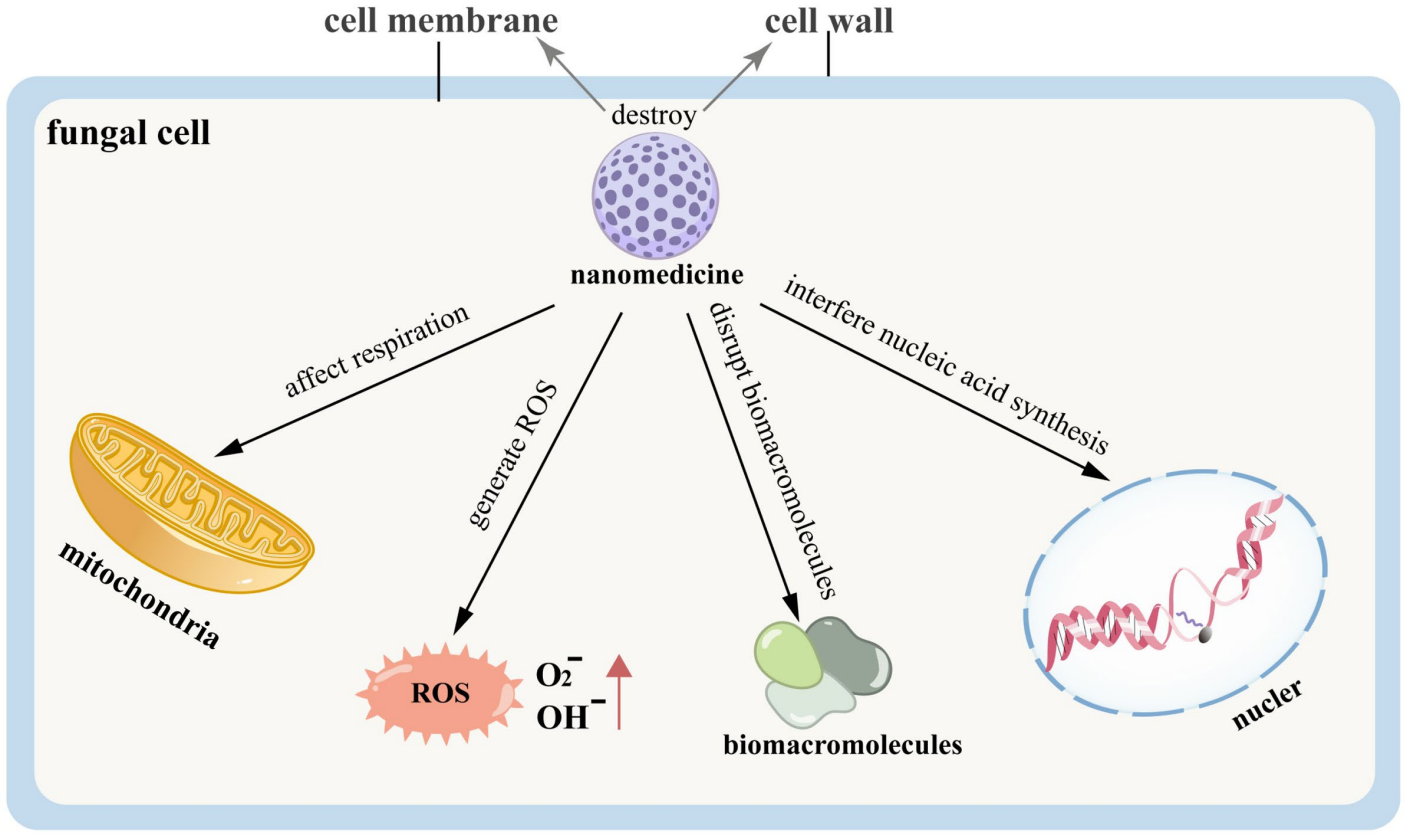
The mechanism of fungal killing by nanomedicine.

Nanomedicine can induce fungal cell death or reduce the expression of mycotoxins by directly disrupting the fungal cell structure, thereby achieving therapeutic effects in the treatment of FK. The related pathogenesis is illustrated in Figure 7. Silver nanoparticles exhibit significant antifungal activity and hold high therapeutic value for FK (Xu et al., 2013). Silver ions can interact with the cell wall and membrane, disrupt intracellular biomacromolecules, affect cellular respiration, generate ROS, interfere with nucleic acid synthesis, and thus inhibit the growth and reproduction of fungi (Shehabeldine et al., 2022; Piecuch et al., 2023). Studies have indicated that AgCu₂O-EDTA nanoparticles (AgCuE NPs) can treat FK caused by *Candida albicans* by destroying the cell wall and membrane of the fungus (Ye et al., 2022). This therapeutic effect may be related to the down-regulation of the Bcr1-related pathway, EFG1, and virulence-related genes, including High Osmolarity Glycerol Response 1 (HOG1) and Checkpoint Kinase 1 (CHK1), which are essential for maintaining the full virulence of *Candida albicans*, along with the up-regulation of oxidative stress-related genes (SOD5, Prx1). The former group is associated with the formation of the cell wall and biofilm of *Candida albicans*, as well as its virulence production and cell adhesion. In contrast, the latter group is linked to the production of ROS, which contribute to fungal cell death (Ye et al., 2022). Scientists have also developed contact lenses loaded with silver nanoparticles (AgNPs), which release these particles to help treat FK (Liu et al., 2018). Hydrogel materials impregnated with silver nanoparticles during the production of contact lenses demonstrate antibacterial properties, reducing the likelihood of microbial keratitis (Fazly Bazzaz et al., 2014). Additionally, silver microspheres (AgMPs) show effective treatment results for candidal keratitis and exhibit biosafety for corneal epithelial cells. Therefore, they can serve as candidate drugs for ocular surface drops aimed at treating FK (Shi et al., 2021).

In addition to silver ions, propolis-based nanofibrous patches (Ulag et al., 2021) and phomopsidione nanoparticle-coated contact lenses can also be utilized to treat corneal microbial keratitis (Bin Sahadan et al., 2019), particularly cases associated with contact lens use. Furthermore, Dectin-1 specific nanobodies have demonstrated a therapeutic effect on *Aspergillus fumigatus* keratitis in murine models, leading to reductions in the mRNA and protein expression levels of IL-1β and IL-6 in human corneal epithelial cells (HCEC) stimulated by infected corneas and *Aspergillus fumigatus*, thereby exerting an anti-inflammatory effect (Liu et al., 2022). Scientists have developed a nanosystem that co-integrates lyticase and gallium ions (MLPGa), which effectively degrades the extracellular polysaccharides found in the cell wall and biofilm of *Candida albicans*, resulting in antifungal activity (He et al., 2022). Moreover, an ultrasmall positively charged carbon dot can penetrate the corneal barrier, open tight junctions, reach the lesion site, and effectively eliminate fungal pathogens (Chen et al., 2024).

Despite the many advantages of nanodrugs in directly treating FK, they still have certain limitations. Nanomaterials have potential toxicity to the human body. For example, silver ions may cause toxic reactions by disrupting cholesterol in cell membranes. Moreover, the metabolism and excretion of some nanomaterials in the body are complex, and their accumulation in tissues may lead to long-term toxicity (Chetoni et al., 2003). Nanoparticles possess metabolic stability, and their metabolism and excretion from the body require the support of the liver and kidneys. Therefore, they may potentially cause damage to liver and kidney functions (Ravindran et al., 2018). Additionally, nanoparticles can affect the activity of enzymes (such as cytochrome P450), thereby influencing the normal metabolism of the body (Ravindran et al., 2018). Additionally, the development of drug resistance in fungal cells and the protective role of biofilms can reduce the effectiveness of nanodrug treatments. The high cost of producing some nanodrugs also limits their large-scale application (Gorantla et al., 2020). Although laboratory studies have shown that nanomaterials have significant therapeutic effects on fungal infections, further research is needed in the academic community to address the challenges in clinical applications, such as individual differences among patients, varying degrees of infection, and the synergistic effects with other treatment methods.

## 7 Indirect treatment

Nanomaterials can be used as drug carriers to increase the residence time of drugs in the cornea and form DDS to improve the treatment efficacy for FK. Additionally, nanomaterials can be utilized to create contact lenses that deliver therapeutic agents for the treatment of FK. Furthermore, nanomaterials can be combined with other treatment modalities, such as photodynamic therapy, to significantly enhance the clinical effectiveness of these approaches.

### 7.1 Drug delivery system

**Figure 8:**
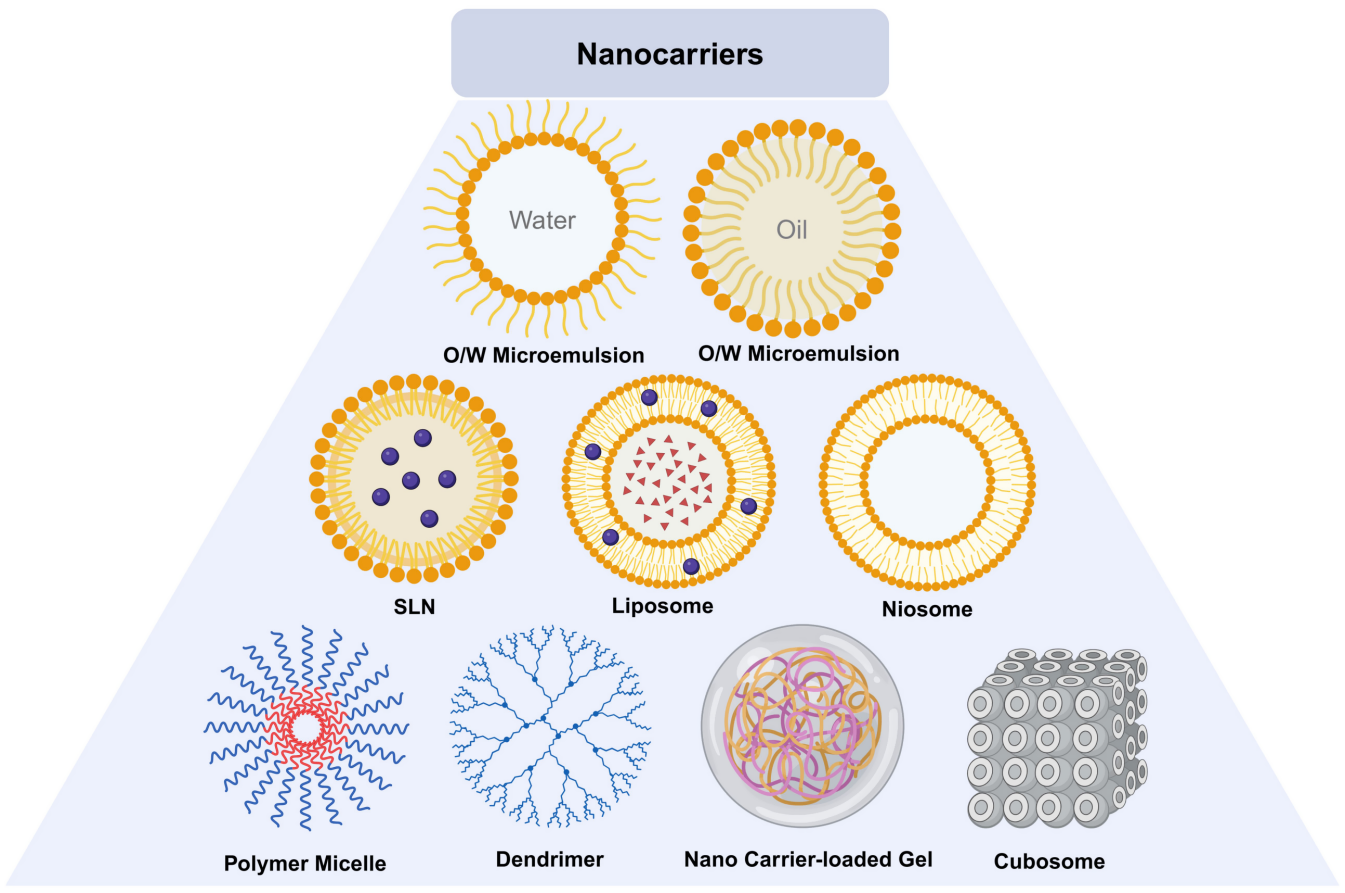
Structure of nanocarriers.

Researchers are exploring effective DDS for FK that exhibit high biocompatibility, excellent drug loading capacity, and efficient drug release capabilities. Nanomaterials such as carbon-based materials, SiO_2_, metal-based materials, and SLNs (Gu et al., 2022) possess characteristics such as small particle size and sustained release, minimizing the need for repeated administration. Additionally, these nanomaterials have a high surface area-to-volume ratio, allowing them to carry significant quantities of drugs. Various ophthalmic DDS have been investigated, including nanoparticles, lipid nanocarriers, microemulsions, liposomes, niosomes, cubosomes, dendrimers, hydrogels, eye inserts, contact lenses, microneedles, carbon quantum dots, and iontophoresis (Polat et al., 2022) (Table 1; Figure 8). The use of ocular DDS to treat FK is currently one of the prominent areas of research.

**Table 1:** Nanomaterial-based ocular DDS and their therapeutic characteristics.

DDS	Diameter	Structure	Molecule type	Advantages	Disadvantages	Residence time of drugs	Drugs encapsulated in the formulation	References
Nanoparticles	< 1 μm	Capsule or spherical	N/A	Exhibiting antifungal activity *in vitro* and reducing inflammation *in vivo*	Low drug loading capacity, particle aggregation, difficult drug release	Higher release in first 8 h vs. NATA, then minimal release	NATA	Gu et al. (2022)
Solid lipid nanoparticles	100–150 nm	Solid lipid nanoparticles	Lipophilic drugs	Higher drug loading capacity, sustained drug releasing, drug targeting and scalable production	Potential cytotoxicity, stringent storage conditions	Up to 8 h	NATA, Amphotericin B, Fluconazole, Voriconazole	Parhi and Suresh (2012); Thukral et al. (2014)
Microemulsion	20–200 nm	Oil–water mixture	Lipophilic or hydrophilic	Enhancing drug residence time on the cornea	May cause eye irritation	Higher permeability and improved antifungal efficacy than drug suspension	Moxifloxacin, Voriconazole	Mohan et al. (2024)
Liposome	<2 μm	Circular vesicles composed of a single or multilayer phospholipid	Lipophilic or hydrophilic	Non-toxic	Difficult to store, high production cost, difficult to transport	N/A	Fluconazole, Voriconazole, Amphotericin B, Posaconazole, Rapamycin	Zhang et al. (2019)
Micelle	<100 nm	Micelle-like	N/A	Simple to manufacture, high drug solubility, low toxicity, long circulation time, tissue permeability	Unstable over long periods, supports only short-term sustained release, insufficient for hydrophilic drugs	Up to 8 h	Amphotericin B, Itraconazole, Voriconazole	Nishiyama and Kataoka (2006); Jaiswal et al. (2015)
Cuboid	48.17 ± 0.65 nm	Cuboid-like	Lipophilic, hydrophilic, or amphiphilic	Non-toxic, biodegradable, high biocompatibility, sustained drug release, high bioadhesion	N/A	N/A	Fluconazole, Sertaconazole nitrate	Nasr et al. (2020) Younes et al. (2018)
Nanocarrier Gel	N/A	Gel network or “egg-box-like”	N/A	Prolonging drug release time, improving efficacy and stability	N/A	Over 12 h	Fluconazole	Patel et al. (2016); Soliman et al. (2019)
Dendrimers	1–10 nm	Dendritic	N/A	Older generations carry cations, suitable for ocular drug delivery	Potential cytotoxicity	N/A	Amphotericin B, Fluconazole, Voriconazole	Kalomiraki et al. (2016)
Niosomes	10–1,000 nm	Vesicle-like	Lipophilic or hydrophilic	Good chemical stability, biodegradable, high biocompatibility, non-immunogenic	N/A	24 h for the release of NATA to reach 75%.	NATA	El-Mofty et al. (2020)

#### 7.1.1 Nanoparticles

Nanoparticles are particles with a diameter of less than 1 μm, which can be rapidly absorbed by cells due to their small size. Nanocarriers can enhance drug penetration, control drug release mechanisms, and act as targeted delivery systems for therapeutic purposes (Sahoo et al., 2008). Some nanoparticles exhibit mucosal adhesion, which prolongs the residence time of drugs in target tissues. When combined with drugs, nanoparticles can form eye drops, significantly improving drug retention and tissue residence time, thereby enhancing patient compliance during treatment (Liu et al., 2012).

The materials used to produce nanoparticles include metals, non-metals, lipids, and polymers. Currently, numerous polymers have been utilized to produce nanoparticles, such as chitosan, alginate, sodium hyaluronate, and gelatin (Gupta et al., 2011; Almeida et al., 2015). Nanoparticles can be classified into nanocapsules and nanospheres. The former encapsulate drugs by forming capsules, while the latter disperse drugs within the matrix material and transport them to the affected area. However, there are still pressing issues, such as low drug loading, particle aggregation, and drug release, that need to be urgently addressed for nanoparticles (Gupta et al., 2010; Nagarwal et al., 2012).

**Figure 9:**
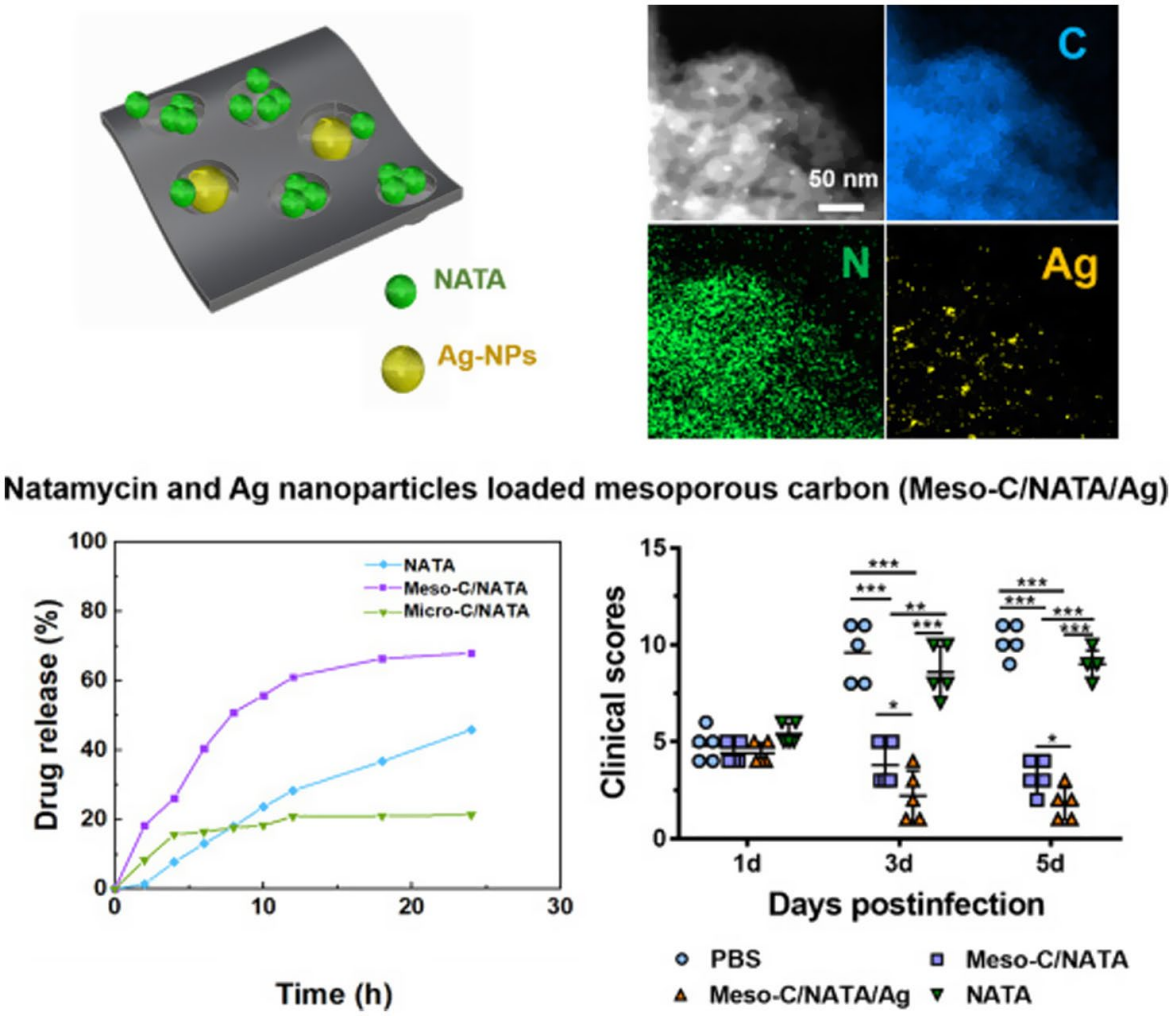
Drug-loaded mesoporous carbon with sustained drug release capacity and enhanced antifungal activity to treat fungal keratitis (Gu et al., 2022).

Gu et al. (2022) utilized drug-loaded mesoporous carbon (Meso-C) containing NATA and silver nanoparticles (Ag NPs) to treat FK, with mesoporous carbon pore sizes ranging from 2 to 50 nm (Figure 9). The drug-loaded mesoporous carbon, prepared using alginate, can remove excess inflammatory factors such as IL-6 and IL-1β (Yushin et al., 2006; Yachamaneni et al., 2010). Meso-C/NATA/Ag NPs exhibit sustained drug release capabilities, enhance antifungal activity, and reduce the inflammatory response. They demonstrate strong antifungal activity *in vitro* and can mitigate the inflammatory response *in vivo*, significantly alleviating corneal inflammation caused by fungi. Unlike Ag NPs, Gu et al. synthesized mesoporous zinc oxide (Meso-ZnO) loaded with NATA for the treatment of Aspergillus FK (Gu et al., 2024). In addition to serving as a drug delivery carrier, Meso-ZnO can also limit fungal growth in a concentration-dependent manner and promote cell migration by activating autophagy, exhibiting anti-inflammatory effects during the fungal infection process. This indicates that Meso-ZnO/NATA is an emerging effective strategy for the treatment of FK.

#### 7.1.2 Lipid nanocarriers

SLNs are solid lipids encapsulated in nanoparticles, composed of physiologically tolerant solid lipids dispersed in a surfactant aqueous solution, typically within the size range of 100–150 nm. They serve as substitutes for classical colloidal carrier systems (Parhi and Suresh, 2012; Thukral et al., 2014). SLNs offer numerous advantages, including high drug loading capacity, sustained drug release, drug targeting, and large-scale production (Khames et al., 2019). The lipophilicity and small particle size characteristics enable SLNs to effectively penetrate biological barriers, while also demonstrating good mucosal adhesion (Singh et al., 2015) and sterilization tolerance (El-Salamouni et al., 2015). However, SLNs may exhibit cytotoxicity and require harsh storage conditions. Researchers have designed nanostructured lipid carriers (NLCs) (Seyfoddin et al., 2010), which encapsulate drugs in solid or liquid lipids. Drugs dissolve in the solid or liquid lipids and are distributed into the aqueous phase of surfactants. The lipid core can prevent drug dissolution, prolong its residence time in the eye, and improve its adsorption onto the tear lipid layer. Additionally, lipid cores can function as penetration enhancers, further enhancing the bioavailability of drugs (Salvi and Pawar, 2019).

Amphotericin B is a widely used drug for treating FK; however, its poor water solubility and systemic toxicity limit its clinical application. Butani et al. (2016) demonstrated that SLNs are effective for the local delivery of amphotericin B by encapsulating it within SLN. Due to local administration and minimal side effects, *in vitro* experiments have confirmed its effectiveness. SLNs enhance the local antifungal effect of the drug while providing sustained drug release. Itraconazole is another antifungal drug used for the treatment of FK. Mohanty et al. (2015) encapsulated itraconazole in SLNs for local delivery to goat corneas, using stearic acid SLNs with higher drug loading than those prepared with palmitic acid. The results showed that the SLNs carrier system enhanced the corneal permeability of itraconazole and exhibited effective inhibition of Aspergillus flavus. Patil et al. (2018) optimized polyethylene glycosylated nanolipid carriers loaded with NATA (NATA PEG NLCs). The optimized NATA PEG NLCs had a small pore size, high drug capture rate, and longer minimum stability time. NATA PEG NLCs demonstrated higher corneal permeability than commercial suspensions, and their therapeutic effect was significantly improved.

#### 7.1.3 Microemulsion

Microemulsion (ME) is a mixture of oil and water, typically prepared with surfactants. It is an opaque liquid, with a particle radius usually ranging from 20 to 200 nm (Tenjarla, 1999). ME can be classified into two types: water-in-oil (W/O) and oil-in-water (O/W), which imparts a certain degree of hydrophilicity or lipophilicity, making it suitable for delivering drugs with either water-based or oil-based components (Lawrence and Rees, 2000). Furthermore, ME can enhance the residence time of drugs in the cornea; however, the surfactants used may cause irritation to the eyes and have adverse effects on patients (Lidich et al., 2019).

Mohan et al. treated FK with a microemulsion of voriconazole, which was prepared using the water titration method. The optimized microemulsion was then coated with chitosan to create a cationic microemulsion. The results indicated that the developed cationic microemulsion exhibited favorable physical and chemical properties, excellent mucosal adhesion, and the ability to continuously release the drug. *In vivo* and *in vitro* experiments demonstrated that, compared to drug suspensions, the cationic microemulsion had higher permeability and a better antifungal effect, thereby providing enhanced therapeutic efficacy (Mohan et al., 2024).

#### 7.1.4 Liposomes

Liposomes are spherical vesicles composed of one or more layers of phospholipids, which are non-toxic and non-degradable (Pietzyk and Henschke, 2000). Due to their amphiphilic nature, phospholipids can encapsulate both lipophilic and hydrophilic drugs. The surface of liposomes may carry charges, and negatively charged liposomes typically release drugs more rapidly than neutral or positively charged liposomes. Considering that the thin mucin layer of corneal epithelial cells carries negative charges, positively charged liposomes may offer greater effectiveness (Hathout et al., 2007). However, liposomes also present challenges such as storage instability, high production costs, and difficulties in transportation, which limit their clinical application (Gorantla et al., 2020).

The application of liposome technology can reduce the toxicity of amphotericin B. It can modulate the rate at which amphotericin B is transferred from the carrier to the cell membrane, which usually leads to decreased drug absorption by fungal target cells. However, this issue can be addressed by increasing the drug dosage. Additionally, liposomes can reduce the toxicity of amphotericin B by adjusting the clearance rate of the complex from the bloodstream (Plotnick, 2000). Zhang et al. (2019) used rapamycin liposomes to treat FK in rats. While rapamycin is only available in systemic formulations and not as an ophthalmic formulation, which limits its clinical use, its lipophilic nature allows for effective encapsulation in phospholipid bilayer liposomes, facilitating the development of ideal ophthalmic formulations. The experiment demonstrated that the group treated with rapamycin liposomes had significantly better therapeutic effects compared to both the control group and the group that did not receive liposomes.

#### 7.1.5 Polymer micelles

Micelles are amphiphilic molecules typically less than 100 nm in size, and they can be categorized into regular micelles and reverse micelles (Trivedi and Kompella, 2010). Micelles offer several significant advantages, including simple preparation, high drug solubility, low toxicity, prolonged drug circulation time, enhanced tissue permeability, and targeted delivery (Nishiyama and Kataoka, 2006). However, traditional micelles tend to be unstable over extended periods, supporting only short-term sustained release, and exhibit less than 100% applicability for hydrophilic drugs (Torchilin, 2007). These limitations necessitate optimization for broader applications. Guo et al. (2020) demonstrated that self-assembled poly (ethylene glycol)-block-poly (glycine methacrylate) (PEG-b-PGMA) micelles carrying NATA show promising results for the treatment of FK. This micelle system facilitates continuous drug release and both *in vivo* and *in vitro* studies have indicated no cytotoxicity by testing with the human corneal epithelial (HCE-2) cell line. PEG shell enhances drug penetration by prolonging micelle contact with the tear mucus layer and promoting corneal uptake. Moreover, the micelles with high biocompatibility achieve controlled drug release via epoxy group hydrolysis. The released drugs exhibit strong antifungal activity, reducing the frequency of administration and enhancing patient medication compliance.

#### 7.1.6 Cubosome

Cubosomes, also known as liquid crystal nanoparticles, are primarily composed of monoglyceride glycerol monoolein (MO) and exhibit a cubic structure. MO is non-toxic, biodegradable, and possesses high biocompatibility. Additionally, cubosomes are capable of continuously releasing drugs and demonstrate high biological adhesiveness (Hartnett et al., 2015).

Nasr et al. (2020) developed a cubic formulation containing fluconazole, which demonstrated a two-fold increase in corneal permeability in rabbits compared to a pure fluconazole solution. This formulation also exhibited improved antifungal efficacy and safety properties in rats. Similarly, Younes et al. (2018) utilized a cubic formulation loaded with sertaconazole nitrate to prevent FK. Their formulation showed excellent mucosal adhesion, extended storage stability, enhanced corneal permeability, and no irritation to the eyes.

#### 7.1.7 Nanocarrier loaded gel

Natural or synthetic polymers could absorb enormous quantities of water and form 3-dimensional crosslinked gels, which is called hydrogel. Nanocarrier-loaded gels are designed for targeted delivery as they can respond to environmental stimuli. Ophthalmic gels, composed of polymers, are particularly susceptible to these environmental influences. The high viscosity of the gel makes it impossible to remove more easily on the surface of the eye, resulting in a longer residence time. The incorporation of nanoparticles into gels effectively addresses the challenges associated with drug release from nanoparticles, extending the duration of drug release (Destruel et al., 2017; Gorantla et al., 2019) and improving both efficacy and stability. Ophthalmic *in situ* gel is composed of environmentally responsive polymers. These polymers will change structurally in response to the environment, such as temperature and pH (Destruel et al., 2017; Gorantla et al., 2019). The drug release of the gel can be changed by changing its porosity or cross-linking level, depending on the polymer used for manufacturing. At the same time, gel can also prevent peptide-drugs from being degraded by enzymes *in vivo*.

Although natural polymers are harmless and biodegradable, they have low physical strength, high variability, and high immunogenicity. Synthetic polymers have better stability, but their biocompatibility and biodegradability are not as good as natural polymers (Van Tomme et al., 2008). The *in situ* gel has another disadvantage. Due to its stability, a higher level of liquid is required in the storage environment to prevent deterioration during storage (Gong et al., 2013).

Morsi et al. (2017) studied the use of ion sensitive in situ gel based on nano emulsion for the delivery of acetazolamide. After the use of surfactants, the results showed that the drugs carried by the gel showed higher stability and stronger therapeutic effect compared with eye drops and oral tablets. Patel et al. (2016) developed a cationic nano-solution-based loteprednol etabonate ophthalmic gel, which demonstrated a bioavailability that is 2.54 times higher than that of the marketed loteprednol etabonate, significantly enhancing the drug’s therapeutic effect.

#### 7.1.8 Dendritic polymers

Dendritic polymers, commonly known as dendrimers, are nanomaterials characterized by dendritic structures consisting of a core, branches, and terminal groups. They have a highly branched structure with a diameter range of 3–20 nm. The core is composed of atoms or molecules, while the branches are connected by covalent bonds. Dendritic polymers have many terminal functional units and internal cavities, which enable them to serve as drug delivery systems. The terminal groups serve as sites for drug attachment (Kalomiraki et al., 2016). Dendritic polymers have strong drug permeability and loading capacity, and can also be targeted for transport to specific cells or tissues. In addition, dendritic polymers have low viscosity and are not easily tangled (Li et al., 2021; Mahaling et al., 2023).

Dendritic polymers exhibit hydrophobic properties. Older generations of dendritic polymers are typically neutral or anionic, making them more suitable for ocular drug applications. In contrast, newer generations often carry cations, which may lead to cytotoxicity and are generally unsuitable for ocular drugs.

Heredero-Bermejo et al. (2020) found that the co administration of carbosilane cationic dendritic polymer molecule BDSQ024 with antifungal drugs caspofungin and amphotericin B resulted in a synergistic decrease in the effective concentration of the drugs, providing a research basis for dendritic polymers as promising biomaterials for studying fungal infections.

#### 7.1.9 Niosome

Niosomes are composed of non-ionic surfactants in aqueous environments and typically range in size from 10 to 1,000 nm (Kazi et al., 2010). They feature a bilayer structure, consisting of both hydrophilic and hydrophobic ends. Similar to liposomes, niosomal vesicles can encapsulate both lipid-soluble and water-soluble drugs (El-Nabarawi et al., 2019). However, niosomes offer better chemical stability and biocompatibility, demonstrating good biological solubility without immunogenicity (Rajpoot, 2019).

El-Mofty et al. (2020) investigated the treatment of FK using ketorolac tromethamine (KETR) gel combined with niosomes loaded with NATA. They found that the drug release time from the niosomes was extended, which they attributed to the presence of cholesterol on the surface of the niosomes. Cholesterol can restrict drug movement, reduce bilayer permeability, and decrease drug efflux, thereby regulating the drug release rate. This modulation results in prolonged drug release times and enhanced therapeutic efficacy.

### 7.2 Enhancing the effectiveness of other treatment methods

Some treatment methods utilize formulations with hydrophilic properties, which can limit their ability to effectively deliver drugs to the cornea. Nanomaterials can enhance these formulations, improving treatment efficacy and serving as adjunctive therapies.

Antimicrobial Photodynamic Therapy (PDT) is an effective antifungal treatment method that employs a specific wavelength of light to activate a photosensitizer. In the presence of oxygen, ROS are generated through the interaction between light and the photosensitizer, which can be lethal to microorganisms (Ozturk et al., 2020). PDT is characterized by high selectivity and minimal damage to surrounding healthy cells. Rose Bangel (RB) is a water-soluble, oxygen-stained anthracene dye that can stain degenerative ocular epithelium, making it useful for examining patients with corneal and conjunctival diseases (Altamirano et al., 2020; El-Kholy et al., 2021). RB is typically activated by 525 nm light (Sztandera et al., 2022). However, due to its water solubility, RB cannot remain in the eye for extended periods (Naranjo et al., 2019). Polymer nanoparticles can encapsulate RB, thereby prolonging drug release and enhancing corneal penetration (Razavi et al., 2022). Ghoniem et al. (2023) utilized Polypyrrole-Gold nanoparticles (AuPpy NPs) containing polypyrrole to carry RB for the treatment of Candida infections in mice. The coupling of RB with AuPpy NPs allows for photothermal and photodynamic effects, resulting in a nanosystem with high loading capacity, excellent dispersion, and enhanced photothermal efficacy. Compared to the use of RB alone, the RB-AuPpy NP combination therapy demonstrates greater effectiveness and may represent a promising new treatment option for FK.

## 8 Conclusion and prospect

Due to the unique anatomical and physiological structure of the eye, conventional drugs often encounter challenges in reaching ocular tissues, resulting in a typically short retention time. This makes the treatment of FK particularly challenging. Nanomaterials have introduced innovative therapeutic strategies for FK, encompassing both direct antimicrobial effects and their application as carriers for indirect treatment. Research on direct treatment has primarily focused on silver nanoparticles, which exhibit significant antifungal properties and demonstrate ocular biocompatibility. In addition, zinc oxide nanoparticles possess a unique mechanism for treating FK, as they can activate autophagy pathways and downregulate inflammatory responses. Gold nanoparticles are used in photodynamic or photothermal therapy for FK, exerting direct effects in FK treatment through photothermal/photodynamic effects. However, due to the relatively enclosed structure of the eye, the complete degradation and clearance pathways of nanomaterials are still under investigation, and the long-term effects of these materials on the human body require careful monitoring. In addition to silver nanomaterials, other polymer-based nanomaterials and co-integrated nanosystems have also shown promising antimicrobial activity. Indirect treatment research has mainly concentrated on utilizing nanomaterials as DDS for ocular administration. These include nanoparticles, lipid-based nanocarriers, microemulsions, liposomes, polymeric micelles, cubosomes, nanocarrier-loaded gels, dendrimers, and vesicles. These systems can achieve targeted drug delivery by prolonging drug release, increasing drug retention time on the cornea, and enhancing therapeutic efficacy. Compared to oral administration or conventional topical eye drops, targeted delivery via nanocarriers is both safer and more effective.

Nanomedicine for FK offers many advantages, such as improving patient compliance, enhancing drug efficacy, and reducing side effects. The emergence of drug delivery systems based on nanocarriers is necessary. These systems will provide a paradigm shift for antifungal treatment by enhancing the solubility and stability of drugs, thereby offering sustained release at the site of infection. Meanwhile, these delivery systems will help reduce systemic exposure and toxicity by increasing therapeutic levels within the corneal tissue, thus improving patient compliance and clinical outcomes. Future research directions are expected to focus on refining these novel designs to make the systems more biocompatible and to enhance their stability. Moreover, there is a need for further exploration of the combination of antifungal agents with these new systems to combat multidrug-resistant fungal strains and reduce the likelihood of treatment failure. However, it is undeniable that the high production costs, challenges in storage and transportation, and certain safety concerns associated with some nanomaterials limit their clinical application. To address these issues, more innovative nanomaterials need to be developed, while existing materials should be improved through optimization of material composition, manufacturing processes, and structural design. It is foreseeable that in the future, more novel or improved nanomaterials will emerge, providing new therapeutic options for FK and advancing their clinical application in FK treatment.

## Glossary

AgCu₂O-EDTA NPs: Silver Copper Oxide-EDTA Nanoparticles
AgNPs: Silver Nanoparticles
AuPpy NPs: Polypyrrole-Gold Nanoparticles
Bcr1: Biofilm Regulatory Protein 1
CHK1: Checkpoint Kinase 1
DDS: Drug Delivery System
EFG1: Enhanced Filamentous Growth Protein 1
FK: Fungal Keratitis
HOG1: High Osmolarity Glycerol 1
IL-1β: Interleukin 1 beta
IL-6: Interleukin 6
ME: Microemulsion
NATA: Natamycin
Niosomes: Non-Ionic Surfactant Vesicles
NLCs: Nanostructured Lipid Carriers
NPs: Nanoparticles
O/W: Oil-in-Water (type of microemulsion)
PDT: Photodynamic Therapy
PEG NLCs: Polyethylene Glycol Nanolipid Carriers
Prx1: Peroxiredoxin 1
RB: Rose Bangel (photosensitizer for PDT)
ROS: Reactive Oxygen Species
SLNs: Solid Lipid Nanoparticles
SOD5: Superoxide Dismutase 5
UFK: Ulcerative Fungal Keratitis
W/O: Water-in-Oil (type of microemulsion)
